# Molecular Recognition Studies on Naphthyridine Derivatives

**DOI:** 10.3390/molecules15031213

**Published:** 2010-03-03

**Authors:** José Carlos Iglesias-Sánchez, Dolores Santa María, Rosa M. Claramunt, José Elguero

**Affiliations:** 1 Departamento de Química Orgánica y Bio-Orgánica, Facultad de Ciencias, UNED, Senda del Rey 9, E-28040 Madrid, Spain; E-Mails: jcisanchez@gmail.com (J.C.I-S.); rclaramunt@ccia.uned.es (R.M.C.); 2 Instituto de Química Médica, CSIC, Juan de la Cierva 3, E-28006, Madrid, Spain; E-Mail: iqmbe17@iqm.csic.es (J.E.)

**Keywords:** host-guest, naphthyridine, biotin, acridine, NMR titrations

## Abstract

The association constants *K_b_* of three hosts **I**–**III** designed to have both enhanced hydrogen bonding donor strength and conformational preorganization with biotin analogues **1**–**5** are reported. ^1^H-NMR titrations under two different concentration conditions have been employed to determine the association constants *K_b_*. A statistical analysis using a presence absence matrix has been applied to calculate the different contributions. Hydrogen bond interactions make naphthyridine derivatives **II** and **III** potent binders and effective receptors for (+)-biotin methyl ester (**1**), due to the complex stabilization by additional hydrogen bonds.

## 1. Introduction

Amide N-*H* groups have been used to produce a wide range of receptors capable of coordinating biologically important molecules and anions [1,2]. General reviews covering anion receptors containing amide groups have been published recently [3,4]. Due to their singular stereoelectronic character, they interact with electron deficient centers through the carbonyl group and with electron rich centers through their N-*H* units; this dual feature has been successfully used for the design of amide-based receptors able to recognize a large variety of guests [1]. On the other hand the recognition capabilities of fused-pyridine and naphthyridine hosts remains an important challenge of supra-molecular chemistry [5].

Our previous works have been focused on the design, synthesis and host-guest behaviour of different receptors using biotin methyl ester (**1**) as model substrate [6,7,8,9,10,11]. Here we turned our attention to modifications of the latter molecule by comparatively studying a series of 4*S*-substituted (3a*R*,6a*S*)-tetrahydro-1*H*-thieno[3,4-d]imidazol-2(3*H*)-ones **2**–**5**. 

## 2. Results and Discussion

**Figure 1:**
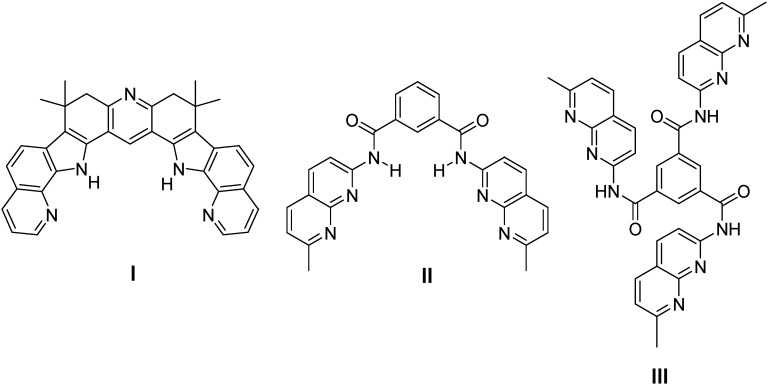
Receptors **I**–**III**.

In this paper we report the measurement and analysis of the binding constants, *K_b_* of five guests **1**–**5** with three receptors, namely 3,4,5,6-tetrahydro-3,3,6,6-tetramethylenebis(pyrido[3,2-g]indolo)[2,3-*a*:3´,2´-*j*]acridine (**I**) [12], *N*,*N’*-bis(7-methyl-1,8-naphthyridin-2-yl)-1,3-benzenedicarboxamide (**II**) [13], and *N*,*N’*,*N’’*-tris(7-methyl-1,8-naphthyridin-2-yl)-1,3.5-benzenetricarboxamide (**III**) [8] (Figure 1). 

**Figure 2:**
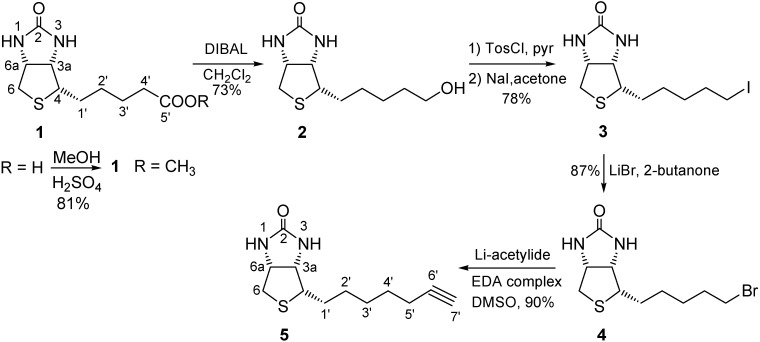
The 4*S*-substituted (3a*R*,6a*S*)-tetrahydro-1*H*-thieno[3,4-*d*]imidazol-2(3*H*)-ones **1**–**5**.

Guests **1** [14] and **2–5** [15] were synthesized according to described procedures with slight variations (Figure 2). The first step was the preparation of biotin methyl ester **1** by acid-catalyzed esterification of biotin. Selective reduction of **1** using DIBAL at −78 ºC afforded alcohol **2** in 73% yield. The iodide **3** was prepared from biotin tosylate by halide substitution with NaI in acetone. The reaction of **3** with LiBr in 2-butanone yielded the formation of bromide **4** in 87% yield. Finally, alkyne **5** was obtained in high yield by the substitution reaction of bromide **4** with lithium acetylide-ethylenediamine in DMSO at 15 ºC.

The ability of receptors containing pyridine or naphthyridine moieties **I**–**III** to recognize and bind the aforementioned guests can be evaluated using ^1^H-NMR spectroscopy. The stoichiometry of the complexes must be determined beforehand to ensure use of the right equations in the titrations.

**Figure 3:**
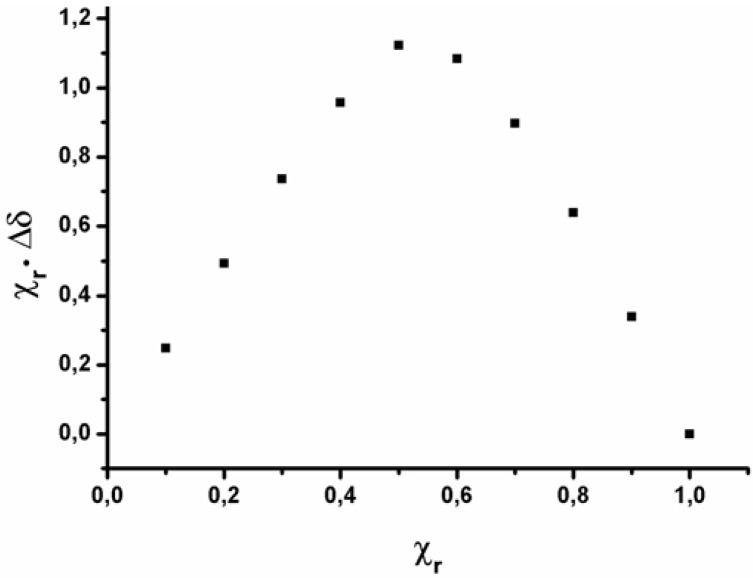
Job plot corresponding to the complexation of host **III** with (+)-biotin methyl ester **1** ([**III**] + [**1**] = 10 mM).

We have used the method of continuous variation to generate Job plots [16] by preparing different mixtures of receptor and guest covering the whole range of molar fractions of the host but keeping constant the total concentration of the solutions (10 mM). The plot of the product between the increment in the chemical shift and the receptor concentration *versus* the molar fraction of the receptor affords a curve. From the value of the maximum, which can be obtained by means of equation *X = m/(m*+*n)* [17], the stoichiometry of the complex is determined. For all the compounds used in this study we always obtained a 1:1 stoichiometry (see Figure 3 for an example).

NMR titrations [18] were perfomed at two different experimental concentrations [6]: the non saturation titration conditions (*nST*) in the 0.2-08 range and the saturation titration conditions (*ST*).

### 2.1. Binding Studies

Association constants *K_b_* for hosts **I**-**III** with biotin derivatives **1**–**5** as guests were determined by ^1^H-NMR titration experiments in CDCl_3_ using the EQNMR software to fit the curves to a 1:1 binding model [19]. The values depicted in Table 1 have been calculated from the chemical shift induced effects of the N-*H* groups of receptors **I**–**III**. 

**Table 1:** Association constants *K_b_* (M^–1^) for **I**–**III** binding **1**–**5** measured at 300 K in CDCl_3_ (Errors ≤ 10%).

	I		II		III	
	*K*_b_^[a]^	*K*_b_^[b]^	*K*_b_^[a]^	*K*_b_^[b]^	*K*_b_^[a]^	*K*_b_^[b]^
**1**	2,700	4,200	67,000	27,200	250,000	148,000
						100^[c]^
**2**	^[d]^	^[d]^	^[d]^	^[d]^	^[d]^	^[d]^
						110^[c]^
**3**	1,500	600	5,600	1,500	800	150
**4**	2,000	630	6,020	1,900	1,700	540
**5**	1,900	244	4,700	1,000	1,000	180

^[a]^ Saturation Titration conditions (*ST*); ^[b]^ non Saturation Titration conditions (*nST*); ^[c]^ with 10% of MeOD; ^[d]^ not detected.

**Figure 4:**
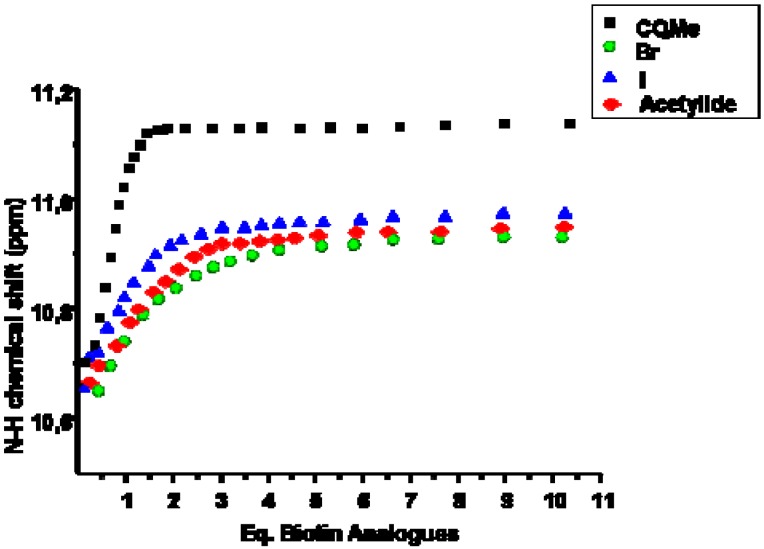
Variations on the chemical shifts of the N-*H* group in host **III** as function of the equivalents of added guest in *ST* conditions.

We have already reported three *K_b_* values of Table 1 using the non Saturation Titration conditions (*nST*): **I·1** 3,800 ± 500 [6], **II·1** 35,000 ± 5,250 [6], and **III·1** 148,000 ± 20,000 [8]. The new values are slightly lower than those determined previously – (0.99 ± 0.04)-fold on average. Addition of biotin analogues **1**–**5** to **I**–**III** in CDCl_3_, results in downfield shifts of the N-*H* groups of receptors **I**–**III** due to host-guest hydrogen bonds interactions. The selectivity displayed by all three receptors **I**–**III** is similar to the trend CO_2_Me >> Br ≥ I ≥ acetylide (Figure 4).

The receptors **II** and **III** have high association constants (6.7 × 10^4^ and 2.5 × 10^5^ M^–1^, respectively) with (+)-biotin methyl ester (**1**), presumably due to the formation of additional hydrogen bonds with the receptors involving the methyl ester that could further stabilize the complex. In addition, the biotin analogues with bulky groups (Br, I and acetylide) will destabilize the complex due to steric repulsion and that seems to be the most important factor influencing the relative poor association constants obtained for these guests.

The biotin analogue **2** was insoluble in CDCl_3_, therefore in order to compare its behavior with that of the other guests, the ^1^H-NMR titration was performed in a 10% MeOD-90% CDCl_3_ solution. As control the (+)-biotin methyl ester (**1**) was titrated under the same conditions as receptor **III**. The obtained association constants *K_b_* were: 100 M^–1^ for **1** and 110 M^–1^ for **2**, suggesting that the latter guest interacts with receptor **III** in a similar manner as it does **1**. On the contrary, guest **2** does not interact with receptors **I** and **II**. 

### 2.2. Data analysis

Finally a more quantitative approach on the association constant values has been attempted. First, we have multiplied by 1.1 (from 100 to 110) all the *K_b_* values of Table 1 for guest **1** (**G1**) to estimate the *K_b_* values of **G2**. Since we have two estimations for *K_b_* we have calculated the regression line between the *ST* and *nST* methods: *ST* = (3,357 ± 2,388) + (1.68 ± 0.04) *nST*, n = 15, R^2^ = 0.992, and used the fitted *ST* values as dependent variables (Table 2). This procedure corresponds to a weighted mixture of both values.

**Table 2:** Absence-presence matrix.

Compound	*K_b_*	HI	HII	HIII	G1	G2	G3	G4	ln *K_b_*
**I·1**	10,447	1	0	0	1	0	0	0	9.25
**II·1**	49,277	0	1	0	1	0	0	0	10.80
**III·1**	253,219	0	0	1	1	0	0	0	12.44
**I·2**	11,156	1	0	0	0	1	0	0	9.32
**II·2**	53,869	0	1	0	0	1	0	0	10.89
**III·2**	278,206	0	0	1	0	1	0	0	12.54
**I·3**	4,370	1	0	0	0	0	1	0	8.38
**II·3**	4,707	0	1	0	0	0	1	0	8.46
**III·3**	3,610	0	0	1	0	0	1	0	8.19
**I·4**	4,420	1	0	0	0	0	0	1	8.39
**II·4**	6,564	0	1	0	0	0	0	1	8.79
**III·4**	4,268	0	0	1	0	0	0	1	8.36
**I·5**	3,769	1	0	0	0	0	0	0	8.23
**II·5**	5,045	0	1	0	0	0	0	0	8.53
**III·5**	3,661	0	0	1	0	0	0	0	8.20

Then we have built a presence/absence matrix (Table 2), known in medicinal chemistry as a Free-Wilson model [20,21]. If we assume that *K_b_* = H × G, then, ln *K_b_* = ln H + ln G. From Table 2 the following contributions can be calculated: ln HI = 7.7, ln HII = 8.5, ln HIII = 9.0, ln G1 = 2.4 and ln G2 = 2.5 (the other G terms are not significant, ln = 0). These values correspond to HI = 2,253, HII = 4,915, HIII = 7,708, G1 = 11.5 and G2 = 12.4 (other G terms = 1).

## 3. Experimental

### 3.1. General

Unless otherwise reported, all reactions were carried out under dry and deoxygenated argon atmospheres. Solvents were freshly distilled and dried before use by standard methods. ^1^H- and ^13^C- NMR spectra were recorded on a Bruker DRX 400 spectrometer (9.4 Tesla, operating at 400.13 MHz for ^1^H and 100.62 MHz for ^13^C, respectively) with a 5-mm inverse detection H-X probe equipped with a z-gradient coil, at 300 K. Chemical shifts (*δ*, in ppm) are given from internal solvent CDCl_3_ (7.26 for ^1^H and 77.0 for ^13^C) and DMSO-*d_6_* (2.49 for ^1^H and 39.5 for ^13^C). 2D gs-COSY (^1^H-^1^H) and 2D inverse proton detected heteronuclear shift correlation spectra [gs-HMQC (^1^H-^13^C) and gs-HMBC (^1^H-^13^C)] were acquired and processed using standard Bruker NMR software and in non-phase-sensitive mode and were carried out to assign the ^1^H and ^13^C signals without ambiguity. 

### 3.2. Synthesis

*Methyl 5-[(3aS,4S,6aR)-2-oxo-hexahydro-1H-thieno[3,4-d]imidazol-4-yl]pentanoate* (**1**) [14]. A suspension of D-(+)-biotin (1g, 4.1 mmol) and H_2_SO_4_ (3 mL) in methanol (20 mL) was heated under reflux for 12 h. Solvents were evaporated under reduced pressure and the residue was poured into ice/water. The precipitated material was filtered, washed with water and dried, to give **1** as a white solid (850 mg, 81%).

*4S-[(3aS,6aR)-5-Hydroxypentyl]tetrahydro-1H-thieno[3,4-d]imidazol-2(3H)-one* (**2**) [15]. Compound **1** (500 mg, 1.93 mmol) was dissolved in CH_2_Cl_2_ (30 mL). The mixture was cooled to −78 ºC and DIBAL (1.0 M, 6.8 mL, 6.78 mmol) was added; the resulting solution was stirred for 2 h at r.t. The mixture was then cooled to -78 ºC and quenched with MeOH. The solvent was evaporated under reduced pressure and the residue was extracted with EtOH using a Soxhlet, to give **2** as a white solid (320 mg, 73%). ^1^H-NMR (DMSO-*d_6_*): *δ* = 6.41 (br. s, 1 H, 3-NH), 6.34 (br. s, 1 H, 1-NH), 4.35 (t, ^3^*J*_5'-H_ = 4.9 Hz, 1 H, OH), 4.29 (br. t, 1 H, 6a-H), 4.12 (br. t, 1 H, 3a-H), 3.35 (m, 2 H, 5'-H), 3.09 (m, 1 H, 4-H), 2.81 (dd, ^2^*J*_Hy_= 12.4 Hz, ^3^*J*_6a-H_ = 5.0 Hz, 1 H, Hx), 2.56 (d, 1 H, Hy), 1.59 (m, 1 H, 1'-H), 1.42 (m, 1 H, 1'-H), 1.40 (m, 2 H, 4'-H), 1.30 (m, 4 H, 2'-H, 3'-H) ppm; ^13^C-NMR (DMSO-*d_6_*): *δ* = 162.8 (CO), 61.1 (C3a), 60.7 (C5'), 59.2 (C6a), 55.6 (C4), 39.9 (C6), 32.3 (C4'), 28.6 (C2'), 28.3 (C1'), 25.6 (C3') ppm.

*4S-[(3aS,6aR)-5-Iodopentyl]tetrahydro-*1*H*-*thieno[3,4-d]imidazol-2(3H)-one* (**3**) [15]. This compound was prepared from biotin tosylate as reported [22]. The biotin tosylate (280 mg, 0.75 mmol) and NaI (216 mg, 0.15 mmol) were stirred in acetone (20 mL) for 24 h. The solvent was removed under reduced pressure and the residue was dissolved in CH_2_Cl_2_ and the organic layer was successively washed with sodium thiosulfate (1.0 N) and water, dried over anhydrous Na_2_SO_4_, and concentrated under vacuum. Purification of the crude material by column chromatography on silica gel with CH_2_Cl_2_/MeOH (5%) gives **3** as a white solid (335 mg, 78%). ^1^H -NMR (CDCl_3_): *δ* = 5.07 (br. s, 1 H, 3-NH), 4.93 (br. s, 1 H, 1-NH), 4.54 (br. t, 1 H, 6a-H), 4.34 (br. t, 1 H, 3a-H), 3.19 (t, ^3^*J*_4'-H_= 6.9 Hz, 2 H, 5'-H), 3.18 (m, 1 H, 4-H), 2.94 (dd, ^2^*J*_Hy_= 12.9 Hz, ^3^*J*_6a-H_ = 5.0 Hz, 1 H, Hx), 2.76 (d, 1 H, Hy), 1.84 (m, 2 H, 4'-H), 1.69 (m, 2 H, 1'-H), 1.46 (m, 4 H, 2'-H, 3'-H) ppm; ^13^C-NMR (CDCl_3_): *δ* = 162.9 (CO), 62.1 (C3a), 60.2 (C6a), 55.4 (C4), 40.6 (C6), 33.1 (C4'), 30.4 (C3'), 28.6 (C1'), 28.0 (C2'), 6.8 (C5') ppm.

*4S-[(3aS,6aR)-5-Bromopentyl]tetrahydro-1H-thieno[3,4-d] imidazol-2(3H)-one* (**4**) [15]. A solution of **3** (800 mg, 2.35 mmol) and LiBr (1.03 g, 11.76 mmol) in 2-butanone (30 mL) was stirred at 80 ºC for 24 h. After cooling at room temperature, 20 mL of 10% NaHSO_3_ were added to quench the reaction. The organic layer was washed with water, dried (MgSO_4_), and evaporated to dryness. The residue was purified by chromatography on silica gel with CH_2_Cl_2_/MeOH (5%) to give **4** as a white solid (595 mg, 87%). ^1^H-NMR (CDCl_3_): *δ* = 5.65 (br. s, 1 H, 3-NH), 5.35 (br. s, 1 H, 1-NH), 4.51 (dd, ^3^*J*_3a-H_ = 7.8 Hz, ^3^*J*_Hx_ = 4.9 Hz 1 H, 6a-H), 4.31 (dd, ^3^*J*_4-H_ = 4.6 Hz, 1 H, 3a-H), 3.41 (t, ^3^*J*_4'-H_ = 6.7 Hz, 2 H, 5'-H), 3.16 (ddd, 1 H, ^3^*J*_1'-H_ = 8.6 Hz, ^3^*J*_1'-H_ = 6.1, 4-H), 2.92 (dd, ^2^*J*_Hy_= 12.8 Hz, ^3^*J*_6a-H_ = 5.0 Hz, 1 H, Hx), 2.75 (d, 1 H, Hy), 1.87 (q, ^3^*J*_3'-H_ = ^3^*J*_5'-H_ = 6.9 Hz, 2 H, 4'-H), 1.69 (m, 2 H, 1'-H), 1.49 (m, 2 H, 3'-H), 1.45 (m, 2 H, 2'-H) ppm;^13^C-NMR (CDCl_3_): *δ* = 163.5 (CO), 62.1 (C3a), 60.1 (C6a), 55.5 (C4), 40.6 (C6), 33.8 (C5'), 32.4 (C4'), 28.5 (C1'), 28.2 (C3' or C2'), 28.1 (C2' or C1') ppm.

*4S-[(3aS,6aR)-Hept-6-ynyl-tetrahydro-1H-thieno[3,4-d]imidazol-2(3H)-one* (**5**) [15]. Lithium acetylide-ethylendiamine complex (194 mg, 2.1 mmol) was suspended in DMSO (5 mL) and cooled to 15 ºC. A solution of **4** (200 mg, 0.68 mmol) in DMSO (3 mL) was added and the mixture was stirred for 1.5 h. The reaction mixture was poured over an ice/brine and the crude material extracted with CH_2_Cl_2_. The organic layer was washed with water, dried (MgSO_4_), and evaporated to dryness. The residue was purified by chromatography on silica gel with CH_2_Cl_2_/MeOH (5%) to give **5** as a white solid (146 mg, 90%). ^1^H-NMR (CDCl_3_): *δ* = 5.02 (br. s, 1 H, 3-NH), 4.95 (br. s, 1 H, 1-NH), 4.51 (dddd, ^3^*J*_3a-H_ = 7.8 Hz, ^3^*J*_Hx_ = 5.1 Hz, ^3^*J*_1-NH_ = ^3^*J*_Hy_ = 1.2 Hz, 1 H, 6a-H), 4.31 (ddd, ^3^*J*_4-H_ = 4.7 Hz, ^3^*J*_3-NH_ = 1.5, 1 H, 3a-H), 3.17 (ddd, 1 H, ^3^*J*_1'-H_ = 8.5 Hz, ^3^*J*_1'-H_ = 6.5 Hz, 4-H), 2.93 (dd, ^2^*J*_Hy_= 12.8 Hz, ^3^*J*_6a-H_ = 5.1 Hz, 1 H, Hx), 2.74 (d, 1 H, Hy), 2.19 (td, ^3^*J*_4'-H_= 6.8 Hz,^ 4^*J*_7'-H_ = 2.7 Hz, 2 H, 5'-H), 1.94 (t, 1 H, 7'-H), 1.68 (m, 2 H, 1'-H), 1.54 (m, 2 H, 4'-H), 1.45 (m, 4 H, 2'-H, 3'-H) ppm; ^13^C-NMR (CDCl_3_): *δ* = 162.9 (CO), 84.4 (C6’), 68.4 (C7'), 62.0 (C3a), 60.1 (C6a), 55.4 (C4), 40.5 (C6), 28.6 (C2'/C3'), 28.5 (C1'), 28.1 (C4'), 18.3 (C5’) ppm.

### 3.3. NMR titrations

Association constants were calculated through ^1^H-NMR titration experiments in CDCl_3_ (and in some cases by adding a 10% MeOD to the CDCl_3_) using the EQNMR software to analyze the results [19]. Changes in the chemical shifts of the N-*H* groups of receptors were used. With respect to the non Saturation Titration conditions (*nST*) we proceeded as described already by ourselves [6,7,8,9]. To perform the measurement of *K*_b_, the host and guest solutions were prepared in a volumetric flask of the appropriate volume (2 mL to 5 mL). 0.5 mL of the host solution **I-III** was taken, put in a NMR tube and successive additions of solutions of guests **1–5** aliquots were made. The number of additions was continued until saturation titration conditions (*ST*) or until reached the 0.8 value of saturation (n*ST*). We present here a typical example of the experimental data for *ST* and n*ST* conditions (Table 3 and Table 4).

**Table 3:** Representative table for a Saturation Titration conditions (*ST*) of host **I** with (+)-biotin methyl ester **1**. Initial [host] = 8.40 × 10^-3^, initial [guest] = 7.78 × 10^-2^.

Volume of added guest (I) (μL)	Total-Volume (μL)	[host I] (10^-3^ M)	[guest 1] (10^-3^ M)	Equivalents of added guest	*δ* (NH) (ppm)
0	500	8.40	0	0	10.7474
5	505	8.32	0.77	0.09268	10.8176
10	510	8.24	1.53	0.18537	10.8729
20	520	8.08	3.00	0.37074	10.9920
30	530	7.93	4.41	0.55611	11.0745
40	540	7.78	5.77	0.74147	11.1484
65	565	7.44	8.96	1.20489	11.2488
90	590	7.12	11.88	1.66832	11.2949
115	615	6.83	14.56	2.13174	11.3352
215	715	5.88	23.42	3.98542	11.4600
315	815	5.15	30.10	5.83910	11.5452
565	1,065	3.94	41.31	10.47332	11.6432
815	1,315	3.19	48.27	15.10753	11.6570
915	1,415	2.97	50.36	16.96121	11.6582

**Table 4:** Representative table for a non Saturation Titration conditions (n*ST*) of host **I** with (+)-biotin methyl ester **1**. Initial [host] = 4.31 × 10^-4^, initial [guest] = 9.24 × 10^-4^.

Volume of added guest (I) (μL)	Total-Volume (μL)	[host I] (10^-4^ M)	[guest 1] (10^-4^ M)	Equivalents of added guest	*δ* (NH) (ppm)
20	520	4.14	0.355	0.08588	10.8521
30	530	4.07	0.523	0.12882	11.0125
40	540	3.99	0.686	0.17176	11.1025
65	565	3.81	1.060	0.27911	11.1730
90	590	3.65	1.410	0.38646	11.2587
115	615	3.50	1.730	0.49380	11.2925
215	715	3.01	2.780	0.92320	11.3014
315	815	2.64	3.580	1.35259	11.3514

## 4. Conclusions

The naphthyridine receptors **II** and **III** exhibit a high selectivity towards the (+)-biotin methyl ester (**1**), due to the stabilization of the complex by additional hydrogen bonds, with an association constant *K_b_* = 6.7 × 10^4^ and 2.5 × 10^5^ M^–1^, respectively, in CDCl_3_. These studies illustrate the capability to modulate the association constant of receptors depending on the biotin type analogue. We have found that the selectivity follows the sequence: CO_2_Me >> Br ≥ I ≥ acetylide.

The simple quantitative model (Table 2) allows to go a step further. The hosts classify in the order: **III** (our host) [8] > **II** (Goswami host) [13] > **I** (Thummel host) [12]. Concerning the guests, the order is **G2** ≥ **G1** ((+)-biotin methyl ester) >> Br ≈ I ≈ acetylide. Although **G2** is insoluble in chloroform, it has an affinity for host **III** slightly better than **G1**. Possibly the terminal OH anchors to the host. This make **G2** a promising candidate for further studies, for instance, modifying the polymethylene chain length.
